# 548 Toward a Burn Risk Calculator

**DOI:** 10.1093/jbcr/irac012.176

**Published:** 2022-03-23

**Authors:** Palmer Q Bessey, Bart D Phillips, Matthew H Phillips, Samuel P Mandell, Callie M Thompson

**Affiliations:** New York-Presbyterian Hospital / Weill Cornell Medicine, New York, New York; BData, Inc, Edina, Minnesota; BData, Inc, Apex, North Carolina; UT Southwestern, Parkland Regional Burn Center, Dallas, Texas; University of Utah Health, Salt Lake City, Utah

## Abstract

**Introduction:**

Risk adjusted statistical modeling of deaths in burns has two major purposes. One is to enable comparison of outcomes between centers (Benchmarking). That requires as precise a model as possible and is applied retrospectively. The other objective is to inform patient and family discussions about prognosis and plans of care. These models are applied prospectively based on limited clinical data. The purpose of this study was to derive a model that could be the basis for such a risk calculator.

**Methods:**

We identified 128,252 records in a national burn registry for initial patient admissions to 103 burn centers between July 2015 through June 2020. Cases from centers with < 100 admissions annually were omitted. We compared a logistic regression model based on the revised Baux score (RBS) (age, burn size, inhalation injury) with a logistic regression model involving age, age^2^, burn size, presence of 3^rd^ degree burn, inhalation injury, respiratory failure, burn etiology, gender, and admission year. We compared the Adjusted R^2^, c statistic and average precision for each model. All calculations were done using CatBoostClassifier in Python.

**Results:**

There were 127,018 patients that served as the basis for these analyses. The RBS model had an Adjusted R^2^ of 0.41 compared with 0.54 for the more detailed model, a c statistic of 0.95 vs 0.98, and an average precision of 0.69 vs 0.76.

**Conclusions:**

Both statistical models of mortality following burn injury demonstrated good accuracy. The model with the most predictor variables had better precision. Both models could serve as useful risk calculators for patients following burn injury.

